# Clinical significance of mTOR, ZEB1, ROCK1 expression in lung tissues of pulmonary fibrosis patients

**DOI:** 10.1186/1471-2466-14-168

**Published:** 2014-10-31

**Authors:** Jong Sun Park, Hyo Jin Park, Young Sik Park, Sang-Min Lee, Jae-Joon Yim, Chul-Gyu Yoo, Sung Koo Han, Young Whan Kim

**Affiliations:** Division of Pulmonary and Critical Care Medicine, Department of Internal Medicine, Seoul National University College of Medicine, Seoul National University Bundang Hospital, Seongnam-si, Gyeonggi-do, Korea; Department of Pathology, Seoul National University Bundang Hospital, Seongnam-si, Gyeonggi-do, Korea; Division of Pulmonary and Critical Care Medicine, Department of Internal Medicine, Seoul National University College of Medicine, Seoul National University Hospital, Medicine, 101 Daehak-ro, Jongno-gu, Seoul, 110-744 Korea

**Keywords:** Pulmonary fibrosis, Immunohistochemical analysis, Usual interstitial pneumonia, mTOR, ZEB1, ROCK1, Survival

## Abstract

**Background:**

Idiopathic pulmonary fibrosis (IPF) is a fatal lung disease of unknown causes. Three proteins (mammalian target of rapamycin, mTOR; zinc finger E-box-binding homeobox 1, ZEB1; Rho-associated, coiled-coil containing protein kinase 1, ROCK1) may be related to pulmonary fibrosis. However, they have not been assessed in human pulmonary fibrosis. We assessed the clinical significance of mTOR, ZEB1, and ROCK1 expression in human pulmonary fibrosis of usual interstitial pneumonia (UIP) pattern.

**Methods:**

The mTOR, ZEB1, and ROCK1 expression was evaluated by immunohistochemical staining of 30 surgical lung biopsy tissues from 26 IPF and 4 UIP pattern connective tissue disease related interstitial lung disease (CTD-ILD) patients. The expression scores correlated with the clinical features.

**Results:**

The mTOR, ZEB1 and ROCK1 mainly expressed in alveolar epithelial cells of UIP lungs. The histological fibrosis scores and lung function decline in the strong mTOR expression group were higher than those in the weak and intermediate expression group. Patients with positive ZEB1 expression had higher fibrosis scores and greater decline in carbon monoxide diffusion capacity (DL_CO_) than patients with negative ZEB1 expression. Patients with positive mTOR or ZEB1 expression had poorer prognosis than that of patients with negative mTOR or ZEB1 expression, although it was not statistically significant. ROCK1 was not associated with the studied clinicopathological features.

**Conclusions:**

The mTOR and ZEB1 expression in pulmonary fibrosis patients significantly correlated with the fibrosis score and lung function decline, indicating that it may be related to the prognosis of pulmonary fibrosis. Further studies involving large numbers of homogeneous IPF patients are warranted.

## Background

Idiopathic pulmonary fibrosis (IPF) is a chronic, progressive, irreversible, lethal lung disease of unknown cause, histologically defined as usual interstitial pneumonia (UIP) [1]. The initiating injury and subsequent pathways for IPF have not been elucidated, but the disease is considered to be an epithelial fibrotic disorder. It is characterized by epithelial injury followed by aberrant wound healing with excessive fibrosis and minimal inflammation. In these processes, growth factors, cytokines, and other mediators are released, followed by excessive extracellular matrix deposit and abnormal mesenchymal cell proliferation in the lungs. However, little is known about the detailed pathogenesis of pulmonary fibrosis development.

Mammalian target of rapamycin (mTOR) complex is a highly conserved intracellular serine/threonine kinase and a major downstream component of phosphatidyl inositol 3-kinase (PI3K). The mTOR regulate translation of specific mRNAs and protein synthesis involved in cell cycle regulation and has been shown to influence tissue fibrosis [2]. A previous study demonstrated that the initiation and progression of pulmonary fibrosis was prevented by the administration of rapamycin, an mTOR inhibitor, to a transgenic mouse with pulmonary fibrosis induced by lung-specific expression of the EGFR ligand, transforming growth factor (TGF)-α [3]. In addition, a previously published case study reported that rapamycin was successfully used to treat a patient with IPF [4].

Zinc finger E-box-binding homeobox 1 (ZEB1) protein is a key transcription factor that acts downstream of TGF-β which has been implicated in the epithelial-mesenchymal transition (EMT) [5]. The EMT is thought to contribute to development of pulmonary fibrosis. Furthermore, there is a report that ZEB1 gene is overexpressed in the lung tissues of interstitial lung disease patients [6].

Rho-associated, coiled-coil containing protein kinase 1 (ROCK1) is a serine/threonine kinase and a downstream effector of Rho. It controls cell adhesion and cell motility via actin-cytoskeleton reorganization and actin-myosin filament bundles regulation. A previous study demonstrated that RhoA/ROCK inhibitor attenuated pulmonary fibrosis in animal models [7].

Therefore, it is hypothesized that mTOR pathway, ZEB1, and ROCK1 may play a role in the pathogenesis of pulmonary fibrosis. However, these proteins have not been assessed in human pulmonary fibrosis. This study was performed to identify the clinical significance of mTOR, ZEB1, and ROCK1 expression in the lung tissues of idiopathic pulmonary fibrosis and UIP pattern connective tissue disease related interstitial lung disease (CTD-ILD) patients.

## Methods

### Study population

We enrolled 30 pulmonary fibrosis patients from January 2005 to May 2010 at Seoul National University Hospital. The study included patients who underwent surgical lung biopsy, and were histologically diagnosed with usual interstitial pneumonia (UIP). The pathological diagnosis was undertaken using previously established criteria, and clinical diagnosis was judged according to the international guidelines [8]. Two normal lung tissues were obtained from certified tissue bank to compare with UIP lungs. The demographic characteristics, pulmonary function, and chest computed tomography (CT) scan images of the study patients were reviewed. The survival data of the patients were obtained from the Ministry of Public Administration and Security. A part of the patient data was reported in a previous study [9]. This study was approved by the Institutional Review Board of the Seoul National University Hospital (H-1105-099-363).

### Immunohistochemical staining

The expression of mTOR, ZEB1, and ROCK1 was evaluated by immunohistochemical staining of formalin-fixed and paraffin-embedded lung tissue sections, which were cut to a thickness of 4 μm. After deparaffinization in xylene and rehydration using graded alcohol to water, these slides were soaked in Target Retrieval Solution (Dako, Carpenteria, CA, USA), and then placed in a water bath at 97°C for 20 min to enable antigen retrieval. The sections were then immersed for 5 min in distilled water containing 3% hydrogen peroxidase to block endogenous peroxidase activity. The sections were incubated for 30 min with primary antibodies for mTOR, ZEB1, and ROCK1 diluted 1:200, 1:100 and 1:50, respectively. The primary rabbit monoclonal mTOR and ROCK1 antibodies were purchased from Abcam (Cambridge, MA, USA). The primary goat polyclonal ZEB1 antibody was obtained from Santa Cruz Biotechnology (Santa Cruz, CA, USA). The Dako EnVision^+^ System that contains a horseradish peroxidase-labeled polymer was used along with LSAB®2 (Dako, Carpenteria, CA, USA), a streptavidin-peroxidase conjugate that serves as the secondary antibody. Routinely processed tissue sections of prostate cancer, breast cancer, and thyroid cancer were used as positive staining controls for mTOR, ZEB1, and ROCK1 staining, respectively. Staining without the primary antibody was also performed to verify staining specificity.

### Histologic analysis

**Figure 1:**
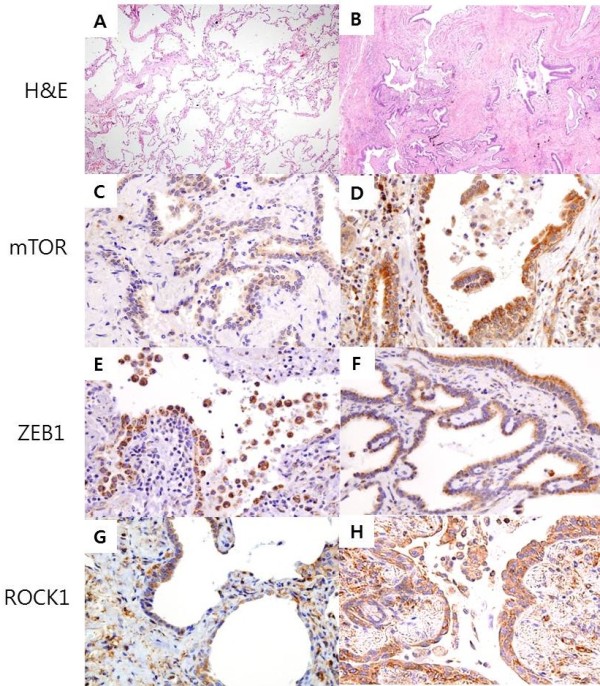
**Representative lung tissue sections of usual interstitial pneumonia patients exemplifying the two principal grades of fibrosis and expression levels observed with the different markers.** Low-power images showing grade of fibrosis (**A**, grade 1 fibrosis; **B**, grade 7 fibrosis; hematoxylin and eosin staining, 40× magnification) The mTOR and ZEB1 expressed in hyperplastic alveolar epithelial cells and some mesenchymal cells from fibrotic areas (**C**, mTOR intermediate; **D**, mTOR strong; **E**, ZEB1 intermediate; **F**, ZEB1 strong). ROCK1 expressed in hyperplastic alveolar epithelial cells, mesenchymal cells, macrophages and lymphocytes of the UIP patients (**G**, ROCK1 intermediate; **H**, ROCK1 strong). **(C-H)**; immunohistochemical staining, brown color, 400× magnification.

Two pathologists reviewed the histology slides independently and without access to the clinical data. The fibrosis score was analyzed using the hematoxylin-and-eosin-stained lung section slides. The extent of fibrotic lesions was scored as 0 (0-10%), 1 (10-25%), 2 (25-50%), 3 (50-75%), or 4 (75-100%). The severity of fibrosis was scored from 0 (normal lung) to 8 (total fibrosis) by determining the average of 10 distinct microscopic fields at 200× magnification in accordance with the Ashcroft scoring system (Figure 1A,B) [10]. Finally, fibrosis scores were calculated by multiplication of the extent of fibrotic lesions to Ashcroft scores. The expression of mTOR, ZEB1, and ROCK1 was scored using both the intensity of staining and the percentage of positively stained alveolar epithelial cells. The total expression score was calculated by multiplying intensity score (0, negative; 1, weak; 2, moderate; 3, strong) to the score of the positively stained cells (0, <10%; 1, 10–49%; 2, 50–75%; 3, >75%). We randomly selected 10 microscopic fields under 100× magnification. Each field was individually assessed and the expression score was determined by obtaining the mean score of all the fields. Finally, the expression level was graded as negative (0), intermediate (1–3), or strong (≥4) based on the total expression score (Figure 1C-H).

### Radiological analysis

Honeycombing on the chest CT scan images was defined as air-filled cysts with thick-walled, between the sizes of several millimeters to several centimeters in diameter. A previously published method [11] was used to assess the honeycombing score. Briefly, the whole lung was divided into 6 lobes (right upper, right middle, right lower, left upper, lingular division, and left lower lobe), and the extent of the honeycombing lesion was assessed as 0 (absent), 1 (0–25%), 2 (25–50%), 3 (50–75%), or 4 (<75%) in each lobe. The honeycombing score was the final sum of all 6 lobes.

### Statistical analysis

The differences in patients’ characteristics, on the basis of the expression of mTOR, ZEB1, and ROCK1, were analyzed by using the Mann-Whitney Test for continuous variables and Fisher’s exact test for categorical variables using SPSS 18.0 statistical software. Pearson’s test was used to verify the association between fibrosis score and mTOR, ZEB1, and ROCK1 expression. A Kaplan-Meier curve was plotted for survival analysis. A p-value of <0.05 was considered as significant.

## Results

### Characteristics of the study patients

The median age of the patients was 57 years (range, 38–74 years), including 14 male patients (46.7%; Table 1). Four patients had connective tissue disease (CTD), however histologic patterns were UIP. Sixteen patients were on steroid or immunosuppressive treatment. The mean forced vital capacity (FVC) and carbon monoxide diffusion capacity (DL_CO_) of the patients was 75% and 65%, respectively. The patient survival rate during the follow-up period (median, 40 months; range, 5–74 months) was 80% (24 patients survived). Baseline clinical characteristics were not different between the patients with IPF and CTD-ILD (Table 2).

**Table 1:** **Characterstics of the study patients**

Variables	N = 30
Age, years, median (range)	57 (38-74)
Male sex	14 (46.7)
Smoking	
Never smoker	19 (63.3)
Former smoker	7 (23.3)
Current smoker	4 (13.3)
Follow up, months	40 (5-74)
Connective tissue disease	4 (13.3)
Arterial blood gas analysis (n = 28)	
pH	7.41 ± 0.0
PaO_2_, mmHg	92.0 ± 16.0
PaCO_2_, mmHg	40.2 ± 4.0
Saturation, %	96.7 ± 1.7
White blood cell count	
Neutrophil, /mm3	4003 ± 1271
Lymphocyte, /mm3	2327 ± 876
Bronchoalveolar lavage (n = 15)	
Alveolar macrophage, %	46.1 ± 21.1
Neutrophil, %	23.0 ± 22.2
Lymphocyte, %	17.5 ± 9.9
Pulmonary function test	
FVC, %	75.3 ± 12.9
FEV_1_, %	86.9 ± 13.5
DL_CO_, %	65.0 ± 25.2
Radiologic features	
Ground glass opacities	20 (66.7)
Reticular density	10 (33.3)
Consolidation	3 (10.0)
Traction bronchiectasis	1 (3.3)
Honeycombing	11 (36.7)
Steroids or immunosuppressive treatment	16 (53.3)
Survivors	24 (80.0)

Definition of abbreviations: FVC = forced vital capacity, FEV_1_ = forced expiratory volume in 1 s, DL_CO_ = carbon monoxide diffusion capacity.

Data are presented as mean ± SD or numbers (%).

**Table 2:** **Comparisons of clinical characteristics between idiopathic pulmonary fibrosis and connective tissue disease related interstitial lung disease of histologic usual interstitial pneumonia pattern**

	IPF	CTD-ILD	p value
	(n = 26)	(n = 4)	
Age, years, median (range)	62 (38-74)	52 (48-55)	0.139
Male sex	14 (53.8)	0	0.066
Ever smoker	11 (42.3)	0	0.141
Pulmonary function test			
FVC, %	73.4 ± 15.6	78.0 ± 15.9	0.746
FEV_1_, %	85.4 ± 16.3	85.0 ± 17.9	0.837
DL_CO_, %	62.8 ± 28.8	65.7 ± 22.2	0.825
Histologic fibrosis score	12.9 ± 8.0	8.6 ± 12.9	0.220
Radiologic honeycombing score	2.1 ± 1.9	1.5 ± 1.9	0.791
mTOR expression score	4.4 ± 2.8	2.7 ± 4.2	0.245
ZEB1 expression score	3.2 ± 2.6	2.5 ± 3.0	0.576
ROCK1 expression score	3.4 ± 2.9	1.7 ± 1.7	0.359
Steroids or immunosuppressive treatment	12 (46.2)	4 (100)	0.089

Definition of abbreviations: IPF = idiopathic pulmonary fibrosis, CTD-ILD = connective tissue disease related interstitial lung disease, FVC = forced vital capacity, FEV_1_ = forced expiratory volume in 1 s, DL_CO_ = carbon monoxide diffusion capacity.

Data are presented as mean ± SD or numbers (%).

### Expression of mTOR, ZEB1 and ROCK1 in the lung tissues of pulmonary fibrosis

**Figure 2:**
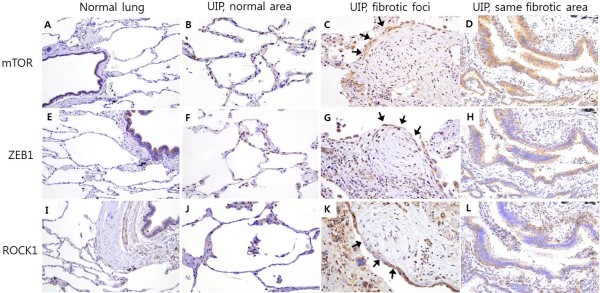
**Representative lung tissue sections from healthy individuals and patients with usual interstitial pneumonia (UIP), stained for mTOR, ZEB1, and ROCK1.** The mTOR, ZEB1 and ROCK1 expression was not detected in alveolar epithelial cells of control normal lung tissue (Figure 2A, E, I, immunohistochemical staining, brown color, 200× magnification). Staining was weak or absent in normal area of UIP lungs (Figure 2B, F, J, 400× magnification). Cells overlying fibroblastic foci showed weak staining, however mesenchymal cells of the fibroblastic foci did not show mTOR, ZEB1 and ROCK1 expression (Figure 2C, G, K; arrows denote location of fibroblastic focus, 400× magnification). Expression of mTOR, ZEB1 and ROCK1 was compared in same fibrotic area (Figure 2D, H, L, 400× magnification).

The mTOR and ZEB1 were mainly expressed in the hyperplastic alveolar epithelial cells and in some mesenchymal cells of UIP lungs (Figure 1C,D,E,F). The ROCK1 expression was detected in the hyperplastic alveolar epithelial cells, some mesenchymal cells, macrophages, and lymphocytes of the UIP patients (Figure 1G,H). The mTOR, ZEB1 expression was not detected in the control normal lung tissue, except in the bronchial epithelial cells (Figure 2A, E). ROCK1 expression was not detected in the normal lung tissue alveolar epithelial cells, but detected in bronchial epithelial cells and surrounding smooth muscle cells (Figure 2I). Cells overlying fibroblastic foci showed weak staining, however mesenchymal cells of the fibroblastic foci did not show mTOR, ZEB1 and ROCK1 expression (Figure 2C, G, K). Staining was weak or absent in normal area of UIP lungs (Figure 2B, F, J).

The correlation of the 3 markers with each other was evaluated (Tables 3, 4 and 5). The mTOR strong expression group showed higher ROCK1 expression scores (p = 0.006); however, ZEB1 expression scores were not related to the mTOR scores (Table 3).

**Table 3:** **Clinical characteristics according to the expression of mTOR**

	mTOR negative,intermediate(n = 13)	mTOR strong(n = 17)	p value
Age, years, median (range)	64 (38-74)	57 (38-70)	0.714
Male sex	5 (38.5)	9 (52.9)	0.484
Ever smoker	4 (30.8)	7 (41.2)	0.708
FVC, %	73.0 ± 15.2	74.8 ± 16.1	0.748
DL_CO_, %	62.1 ± 20.3	64.1 ± 32.7	0.857
PaO_2_, mmHg	99.8 ± 18.1	86.2 ± 11.6	0.023
BAL Neutrophil, %	25.5 ± 24.5	21.3 ± 21.9	0.736
BAL Lymphocyte, %	22.5 ± 12.9	14.2 ± 6.0	0.116
Radiologic honeycombing score	1.7 ± 2.7	2.4 ± 2.6	0.351
Histologic fibrosis score	10.1 ± 8.7	15.0 ± 8.1	0.034
Δ FVC, mL	109 ± 375	-151 ± 174	0.044
Δ DL_CO_, mL/mmHg/min	0.72 ± 2.24	-1.19 ± 1.36	0.023
Δ FVC, %	6.7 ± 17.1	- 4.4 ± 4.9	0.041
Δ DL_CO_, %	5.2 ± 12.9	- 5.2 ± 6.2	0.021
Survivors	11 (84.6)	13 (76.5)	0.672
ZEB1	2.4 ± 2.4	3.7 ± 2.8	0.185
ROCK1	1.6 ± 1.9	4.4 ± 2.9	0.006

Definitions of *abbreviations:* BAL = bronchoalveolar lavage.

Δ: change of pulmonary function per year (final minus initial pulmonary function).

Data are presented as mean ± SD or numbers (%) and analyzed by the Fisher’s exact test or Mann-Whitney test.

**Table 4:** **Clinical characteristics according to the expression of ZEB1**

	ZEB1 negative(n = 4)	ZEB1 intermediate,strong(n = 26)	p value
Age, years, median (range)	61 (52-74)	57 (38-72)	0.362
Male sex	1 (25.0)	13 (50.0)	0.602
Ever smoker	2 (50.0)	9 (34.6)	0.611
FVC, %	86.0 ± 17.5	72.2 ± 14.7	0.099
DL_CO_, %	61.5 ± 19.7	63.5 ± 29.1	0.892
PaO_2_, mmHg	88.1 ± 5.5	92.7 ± 17.1	0.606
BAL Neutrophil, %	37.6 ± 31.6	19.3 ± 19.3	0.213
BAL Lymphocyte, %	13.3 ± 5.6	18.5 ± 10.6	0.433
Radiologic honeycombing score	1.5 ± 3.0	2.1 ± 2.6	0.656
Histologic fibrosis score	1.6 ± 1.3	14.0 ± 8.0	<0.001
Δ FVC, mL	105 ± 487	- 47 ± 298	0.517
Δ DL_CO_, mL/mmHg/min	2.16 ± 3.54	- 0.57 ± 1.77	0.067
Δ FVC, %	4.8 ± 17.6	0.2 ± 13.0	0.647
Δ DL_CO_, %	14.0 ± 21.5	- 1.8 ± 9.1	0.047
Survivors	4 (100)	20 (76.9)	0.557
mTOR	1.5 ± 3.0	4.6 ± 2.8	0.039
ROCK1	1.3 ± 0.9	3.5 ± 2.9	0.183

Definitions of *abbreviations:* BAL = bronchoalveolar lavage.

Δ: change of pulmonary function per year (final minus initial pulmonary function).

Data are presented as mean ± SD or numbers (%) and analyzed by the Fisher’s exact test or Mann-Whitney test.

**Table 5:** **Clinical characteristics according to the expression of ROCK1**

	ROCK1 negative,intermediate(n = 16)	ROCK1 strong(n = 14)	p value
Age, years, median (range)	63 (39-74)	55 (38-72)	0.110
Male sex	7 (43.8)	7 (50.0)	1.000
Ever smoker	6 (37.5)	5 (35.7)	1.000
FVC, %	73.6 ± 13.6	74.5 ± 17.9	0.871
DL_CO_, %	58.4 ± 21.7	68.8 ± 33.2	0.332
PaO_2_, mmHg	92.8 ± 12.3	91.1 ± 19.9	0.784
BAL Neutrophil, %	32.4 ± 26.8	14.7 ± 14.3	0.129
BAL Lymphocyte, %	18.8 ± 12.5	16.3 ± 7.6	0.646
Radiologic honeycombing score	1.7 ± 2.7	2.4 ± 2.6	0.496
Histologic fibrosis score	10.1 ± 8.7	15.0 ± 8.1	0.123
Δ FVC, mL	- 20 ± 190	- 45 ± 401	0.856
Δ DL_CO_, mL/mmHg/min	- 0.05 ± 1.72	- 0.59 ± 2.33	0.547
Δ FVC, %	0.4 ± 6.3	0.8 ± 17.8	0.936
Δ DL_CO_, %	1.0 ± 10.3	-1.9 ± 11.9	0.530
Survivors	12 (75.0)	12 (85.7)	0.657
mTOR	3.1 ± 2.9	5.4 ± 2.7	0.033
ZEB1	2.9 ± 2.9	3.4 ± 2.3	0.361

Definitions of *abbreviations:* BAL = bronchoalveolar lavage.

Δ: change of pulmonary function per year (final minus initial pulmonary function).

Data are presented as mean ± SD or numbers (%) and analyzed by the Fisher’s exact test or Mann-Whitney test.

### Clinical characteristics in relation to the expression of mTOR

**Figure 3:**
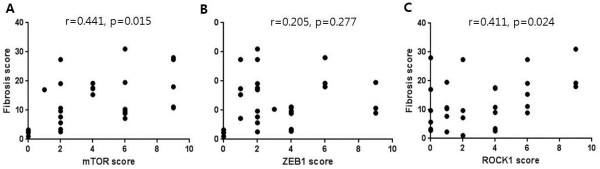
**Association of mTOR(A), ZEB1(B), and ROCK1(C) expression with fibrosis score.**

**Figure 4:**
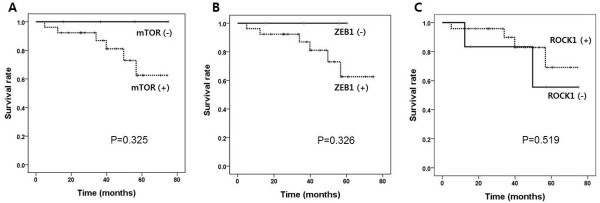
**Patient survival in association with the expression of mTOR(A), ZEB1(B) and ROCK1(C) expression; (-) represents negative expression group and (+) represents intermediate and strong expression group.**

The strong mTOR expression group had significantly higher (p = 0.034) histological fibrosis scores than those of the negative and intermediate expression groups (Table 3). In addition, the annual decline in FVC and DL_CO_ was higher in the strong mTOR expression group than that in the negative and intermediate expression groups (Table 2). PaO_2_ was lower in strong expression group than in the negative and intermediate expression groups (p = 0.023). The strong mTOR expression group had significantly higher ROCK1 expression scores than those of the negative and intermediate expression groups. The mTOR expression score significantly correlated with the fibrosis score (r = 0.441, p = 0.015; Figure 3A). However, mTOR expression did not correlate with the honeycombing score, FVC, and DLCO. During the study period, no fatality was observed in the mTOR-negative group. In contrast, the mTOR-positive (intermediate and strong) group had 6 fatalities. (Figure 4A; p = 0.325).

### Clinical characteristics in relation to ZEB1 expression

Patients with positive (intermediate and strong) ZEB1 expression showed higher fibrosis scores, greater annual rate of decline in DL_CO_ (% predicted), and higher mTOR scores than that of patients with negative ZEB1 expression (Table 4). The ZEB1 expression score did not significantly correlate with the fibrosis score (Figure 3B), honeycombing score, FVC, and DLCO. Similar to the findings related to mTOR lung tissue expression, no fatality was observed in the ZEB1-negative group (Figure 4B; p = 0.326).

### Clinical characteristics in relation to ROCK1 expression

Pulmonary function, honeycombing, and histological fibrosis scores were not different among ROCK1 negative, intermediate, and strong expression groups (Table 5). However, ROCK1 expression was associated with fibrosis score (r = 0.441, p = 0.024; Figure 3C). Survival was not significantly associated with the ROCK1 expression (Figure 4C).

## Discussion

This was the first study to evaluate mTOR, ZEB1, and ROCK1 expression in the lung tissues of pulmonary fibrosis patients. We evaluated the expression of mTOR, ZEB1, and ROCK1 proteins in the lung tissue, along with clinical parameters such as radiological honeycombing score, histological fibrosis score, pulmonary function, and patient survival rate. The expression of mTOR, ZEB1, and ROCK1 was increased in most of the lung tissues of pulmonary fibrosis patients. The expression of mTOR and ZEB1 significantly correlated with the fibrosis score and pulmonary function change. During the follow-up period, there were no fatalities in the patient group with undetectable mTOR or ZEB1 expression. However, ROCK1 expression was not associated with the assessed clinicopathologic features.

In clinical settings, mTOR inhibitors are used as immunosuppressants to prevent graft rejection. Activated mTOR complex regulates cell cycle and therefore, treatment with mTOR inhibitors, such as rapamycin, leads to cell cycle arrest in the G1 phase. The key immunosuppression mechanism of mTOR inhibitors is to block clonal proliferation and expansion of stimulated lymphocytes. However, mTOR inhibitors also block proliferation of other cell types such as vascular smooth muscle cells, mesangial cells, and endothelial cells and have anti-fibrotic activity [12]. The anti-fibrotic effects of mTOR inhibition have been reported in various rat models of diabetic nephropathy, glomerulosclerosis, tubulointerstitial fibrosis, and liver cirrhosis [13–16]. In the murine pulmonary fibrosis model, rapamycin treatment resulted in reduced lung collagen deposition [3, 17, 18]. However, the role of mTOR in human pulmonary fibrosis has not been evaluated.

In our study, expression of mTOR was increased in most of the UIP lung tissues and significantly correlated with the fibrosis score and pulmonary function change. Thus, mTOR expression may be related to the prognosis of pulmonary fibrosis. mTOR was mainly expressed in the alveolar epithelial cells of pulmonary fibrosis patients. The alveolar epithelial cells have a crucial role in pathogenesis of IPF. Repetitive microinjuries to the alveolar epithelium drive a pathogenic cascade of IPF. Activated alveolar epithelial cells stimulate fibroblasts to secrete various profibrotic cytokines [19]. mTOR expression in the alveolar epithelial cells indicates that mTOR may play a role in the pathogenesis of IPF by promoting alveolar epithelial cell proliferation. Although mesenchymal cells such as fibroblasts or myofibroblasts rarely expressed mTOR in our study, a previous study showed that an mTOR inhibitor suppressed fibroblast growth [20]. Recently, it was reported that rapamycin suppressed TGF-β1-induced expression of collagen and fibronectin levels in primary human lung fibroblasts [21]. The role of mTOR should be further evaluated not only in fibroblasts but also in alveolar epithelial cells.

Considering the antiproliferative effect of the mTOR inhibitors [12] and expression of mTOR in UIP lungs, mTOR inhibitor may be a potential therapeutic drug for the treatment of IPF. The effectiveness of rapamycin (sirolimus) for IPF is currently under investigation in a pilot clinical trial [22].

Epithelial-mesenchymal transition (EMT) induced by TGF-β is thought to be a potential mechanism underlying the development of pulmonary fibrosis. TGF-β induces EMT by both Smad-dependent and Smad-independent signaling events [23]. Smad-dependent signaling up-regulates the expression of several transcription factors, including Snail, Slug, Twist, and members of the ZFH family, ZEB1 and ZEB2, all of which are essential for EMT induction. These transcription factors activate EMT by binding to the E-box elements present in the E-cadherin promoter and by suppressing the synthesis of E-cadherin, a cell-cell adhesion protein [24]. A recent study revealed that the ZEB1 gene is overexpressed in the lung tissues from patients with interstitial lung disease [6]. In our study, ZEB1 was overexpressed in the alveolar epithelial cells from pulmonary fibrosis patients. Moreover, ZEB1 expression was related to fibrosis score and change in DL_CO_.

In addition to Smad pathway, multiple signaling proteins have been implicated in the TGF-β-induced EMT. ROCK is one of the proteins associated with EMT [25]. ROCK inhibitor with ZEB1/ZEB2 knockdown restored epithelial proteins from the mesenchymal state of renal tubular epithelial cells [26]. A previous study reported that treatment with ROCK inhibitor reduced the fibrosis score in a murine lung fibrosis model [7]. However, ROCK1 expression was not associated with any other clinicopathological characteristics in our study.

Further studies are required to address the findings of our data. For examples, functional studies are needed to identify the role of mTOR, ROCK1, and ZEB1 in the pathogenesis of pulmonary fibrosis. In addition, we included CTD-ILD patients that showed UIP features in lung tissue. At enrollment, these patients were diagnosed with IPF. However, the diagnosis was changed to CTD-ILD on follow-up visits due to the late presentation of CTD symptoms. It is possible that the marker expression were influenced by the inclusion of other disease types (CTD-ILD) that were not IPF. However, baseline lung function and expression scores of each marker were not different in patients with IPF and CTD-ILD. After excluding 4 patients with CTD-ILD, change of lung function decline and fibrosis scores were related to the mTOR and ZEB1 expression level (data not shown).

Furthermore, a study with a large sample size is required to evaluate whether the association of patient survival rate with the expression level of mTOR and ZEB1 is statistically significant.

## Conclusions

We first demonstrated that mTOR, ZEB1 and ROCK1 was expressed in hyperplastic alveolar epithelial cells of human pulmonary fibrosis lungs. Furthermore, mTOR and ZEB1 were associated with clinical outcomes such as lung function decline. Thus, mTOR and ZEB1 expression may be related with the prognosis of pulmonary fibrosis and disease progression. Because our patients included some patients with CTD-ILD, further studies involving large numbers of homogeneous IPF patients are warranted to determine whether mTOR and ZEB1 are true prognostic markers of pulmonary fibrosis.

## Abbreviations

mTOR: mammalian target of rapamycin
ROCK1: Rho-associated, coiled-coil containing protein kinase 1
UIP: Usual interstitial pneumonia
ZEB1: Zinc finger E-box-binding homeobox 1.
